# Generalized Ulam-Hyers Stability, Well-Posedness, and Limit Shadowing of Fixed Point Problems for **α**-**β**-Contraction Mapping in Metric Spaces

**DOI:** 10.1155/2014/569174

**Published:** 2014-01-23

**Authors:** Wutiphol Sintunavarat

**Affiliations:** Department of Mathematics and Statistics, Faculty of Science and Technology, Thammasat University Rangsit Center, Pathum Thani 12121, Thailand

## Abstract

We study the generalized Ulam-Hyers stability, the well-posedness, and the limit shadowing of the fixed point problem for new type of generalized contraction mapping, the so-called **α**-**β**-contraction mapping. Our results in this paper are generalized and unify several results in the literature as the result of Geraghty (1973) and the Banach contraction principle.

## 1. Introduction and Preliminaries

The stability problem of functional equations, first initial from a question of Ulam [1] in 1940, concerns the stability of group homomorphisms. In next year, Hyers [2] first gives some partial answer of Ulam's question for Banach spaces and then this type of stability is called the Ulam-Hyers stability. This opened an avenue for further study and development of analysis in this field. Subsequently, many researchers have studied and extended Ulam-Hyers stability in many ways. In particular, there are a number of results that studied and extended Ulam-Hyers stability for fixed point problems as Bota et al. [3], Bota-Boriceanu and Petrusel [4], Brzdȩk et al. [5], Brzdek and Cieplinski [6, 7], Cadariu et al. [8], Lazǎr [9], Rus [10], and F. A. Tise and I. C. Tise [11].

On the other hand, the notion of well-posedness and limit shadowing property of a fixed point problem have evoked much interest to many researchers, for example, De Blassi and Myjak [12], Reich and Zaslavski [13], Lahiri and Das [14], and Popa [15, 16].

Recently, Samet et al. [17] introduced the following concept.

**Definition 1 (see [17])**

Let *X* be a nonempty set and *α* : *X* × *X* → [0, *∞*) be a mapping. A mapping *f* : *X* → *X* is said to be *α*-admissible if it satisfies the following condition:

(1) for  x,y∈X for  which  α(x,y)≥1⟹α(f(x),f(y))≥1.

**Example 2**

Let *X* = (0, *∞*). Define *f* : *X* → *X* and *α* : *X* × *X* → [0, *∞*) by *f*(*x*) = 2^*x*^ for all *x* ∈ *X* and

(2) $$\begin{array}{c} \alpha \left(x,y\right)=\{\begin{array}{cc} e^{\left|\sin x+\cos y\right|}, & x\geq y,\\ e^{x-y}, & x<y. \end{array} \end{array}$$

Then, *f* is *α*-admissible.

**Example 3**

Let *X* = [1, *∞*). Define *f* : *X* → *X* and *α* : *X* × *X* → [0, *∞*) by

(3) $$\begin{array}{c} f\left(x\right)=\{\begin{array}{cc} \frac{x+2}{2}, & x\in \left[1,2\right]\\ \ln x, & x\in \left(2,\infty \right), \end{array}\\ \alpha \left(x,y\right)=\{\begin{array}{cc} \frac{{\left(x+y\right)}^{2}}{2}, & x,y\in \left[1,2\right]\\ \frac{1}{1+\min\left\{x,y\right\}}, & \text{otherwise}. \end{array} \end{array}$$

Then, *f* is *α*-admissible.

**Remark 4**

Every nondecreasing self-mapping *f* is *α*-admissible.

Samet et al. [17] established fixed point theorems for some type of generalized contraction mapping by using the concept of *α*-admissible mapping. Also, they applied these results to derive fixed point theorems in partially ordered metric spaces. As application, they studied the ordinary differential equations via the main results. Several researchers studied and improved contraction mappings via the concept of *α*-admissible mapping in metric spaces and other spaces (see [18–22] and references therein).

The first aim of this work is to introduce new type of contraction mapping which generalized several types of mappings in the literature as Geraghty-type contraction mapping [23] and Banach contraction mapping [24]. Also, we establish some existence and uniqueness of fixed point theorems for such mappings in metric spaces by using the concept of *α*-admissible mapping. Our second purpose is to present generalized Ulam-Hyers stability, well-posedness, and limit shadowing of fixed point problems for this mapping in metric spaces.

## 2. Main Results

Let *Υ* denote the class of all functions *β* : [0, *∞*)→[0,1) which satisfies the following condition.

For any sequence {*t*_*n*_} of nonnegative real numbers, we have

(4) $$\begin{array}{c} \underset{n\to \infty }{\lim}\beta \left(t_{n}\right)=1\;\text{implying}\;\text{that}\;\underset{n\to \infty }{\lim}t_{n}=0. \end{array}$$

This class is first introduced by Geraghty [23] in 1973. Afterwards, there are many results about fixed point theorems by using such function in this class in many spaces with different contractions; for details we refer the readers to [25–28] and references therein.

The following are examples of some functions in *Υ*.

(i) Consider *β*_1_(*t*) = *k* for all *t* ∈ [0, *∞*), where *k* ∈ [0,1).
(ii) Consider
  (5) $$\begin{array}{c} \beta_{2}\left(t\right)=\{\begin{array}{cc} \frac{\ln\left(1+t\right)}{t}; & t>0,\\ r\in \left[0,1\right); & t=0. \end{array} \end{array}$$

First we give the following definition as a generalization of Banach contraction mappings.

**Definition 5**

Let (*X*, *d*) be a metric space and *f* : *X* → *X* a given mapping. One says that *f* is an *α*-*β*-contraction mapping if there exist two functions *α* : *X* × *X* → [0, *∞*) and *β* ∈ *Υ* such that

(6) $$\begin{array}{c} {\left[\alpha \left(x,y\right)-1+\delta_{*}\right]}^{d(f(x),f(y))}\leq \delta^{\beta (d(x,y))d(x,y)}, \end{array}$$

for all *x*, *y* ∈ *X*, where 1 < *δ* ≤ *δ*_∗_.

**Remark 6**

It is easy to check that an *α*-*β*-contraction mapping reduces to a Geraghty-type contraction mapping if *α*(*x*, *y*) = 1 for all *x*, *y* ∈ *X*.

Next, we introduce the transitive mapping which is useful for our main result.

**Definition 7**

Let *X* be a nonempty set. A mapping *α* : *X* × *X* → [0, *∞*) is called transitive if it satisfies the following condition:

(7) for  x,y,z∈X for  which  α(x,y)≥1, α(y,z)≥1⟹α(x,z)≥1.

Our first main result is the following.

**Theorem 8**

Let (*X*, *d*) be a complete metric space and *f* : *X* → *X* an *α*-*β*-contraction mapping satisfying the following conditions:

- *f* is *α*-admissible;
- *α* is transitive;
- there exists *x*_0_ ∈ *X* such that *α*(*x*_0_, *f*(*x*_0_)) ≥ 1;
- *f* is continuous.

Then the fixed point problem of *f* has a solution; that is, there exists x* ∈ *X* such that x* = *f*(x*).

**Proof**

Let *x*_0_ ∈ *X* such that *α*(*x*_0_, *f*(*x*_0_)) ≥ 1 (such a point exists from condition (iii)). Define the sequence {*x*_*n*_} in *X* by

(8) $$\begin{array}{c} x_{n}=f\left(x_{n-1}\right)\;\forall n\in \mathbb{N}. \end{array}$$

If *x*_*n*_ = *x*_*n*−1_ for some *n* ∈ *ℕ*, then *x*_*n*_ = *f*(*x*_*n*_); that is, *x*_*n*_ is a fixed point of *f* and thus the proof ends. Therefore, we may assume that

(9) $$\begin{array}{c} x_{n}\neq x_{n-1}\;\forall n\in \mathbb{N}. \end{array}$$

Since *f* is *α*-admissible and *α*(*x*_0_, *x*_1_) = *α*(*x*_0_, *f*(*x*_0_)) ≥ 1, we get *α*(*x*_1_, *x*_2_) = *α*(*f*(*x*_0_), *f*(*x*_1_)) ≥ 1. By induction, we get

(10) $$\begin{array}{c} \alpha \left(x_{n-1},x_{n}\right)\geq 1\;\forall n\in \mathbb{N}. \end{array}$$

For *n* ∈ *ℕ*, we have

(11) δd(xn,xn+1)=δd(f(xn−1),f(xn))≤δ∗d(f(xn−1),f(xn))≤[α(xn−1,xn)−1+δ∗]d(f(xn−1),f(xn))≤δβ(d(xn−1,xn))d(xn−1,xn).

This implies that

(12) $$\begin{array}{c} d\left(x_{n},x_{n+1}\right)\leq \beta \left(d\left(x_{n-1},x_{n}\right)\right)d\left(x_{n-1},x_{n}\right)<d\left(x_{n-1},x_{n}\right) \end{array}$$

for all *n* ∈ *ℕ*. Therefore, the sequence {*d*(*x*_*n*−1_, *x*_*n*_)} is strictly decreasing and so *d*(*x*_*n*−1_, *x*_*n*_) → *d* as *n* → *∞* for some *d* ≥ 0. Next, we claim that *d* = 0. Assume on the contrary that *d* > 0. On taking limit as *n* → *∞* in (12), we obtain that

(13) $$\begin{array}{c} \underset{n\to \infty }{\lim}\beta \left(d\left(x_{n-1},x_{n}\right)\right)=1. \end{array}$$

Since *β* ∈ *Υ*, we have lim⁡_*n*→*∞*_⁡*d*(*x*_*n*−1_, *x*_*n*_) = 0, which is a contradiction. Therefore, *d* = 0 and thus

(14) $$\begin{array}{c} \underset{n\to \infty }{\lim}d\left(x_{n-1},x_{n}\right)=0. \end{array}$$

Next, we show that {*x*_*n*_} is a Cauchy sequence. On the contrary, assume that {*x*_*n*_} is not a Cauchy sequence. Then there exists *ϵ* > 0 and subsequence of integers *n*_*k*_ and *m*_*k*_ with *n*_*k*_ > *m*_*k*_ ≥ *k* such that

(15) $$\begin{array}{c} d\left(x_{m_{k}},x_{n_{k}}\right)\geq \epsilon \end{array}$$

for all *k* ∈ *ℕ*. Further, corresponding to *m*_*k*_, we can choose *n*_*k*_ in such a way that it is the smallest integer with *n*_*k*_ > *m*_*k*_ ≥ *k* and satisfying (15). Then we have

(16) $$\begin{array}{c} d\left(x_{m_{k}},x_{n_{k}}\right)\geq \epsilon ,\\ d\left(x_{m_{k}},x_{n_{k}-1}\right)<\epsilon . \end{array}$$

From (16) and the triangle inequality, we have

(17) ϵ≤d(xmk,xnk)≤d(xmk,xnk−1)+d(xnk−1,xnk)<ϵ+d(xnk−1,xnk).

Letting *k* → *∞* and using (14), we have

(18) $$\begin{array}{c} \underset{k\to \infty }{\lim}d\left(x_{m_{k}},x_{n_{k}}\right)=\epsilon >0. \end{array}$$

Since *α* is transitive and *n*_*k*_ > *m*_*k*_, we can conclude that

(19) $$\begin{array}{c} \alpha \left(x_{m_{k}},x_{n_{k}}\right)\geq 1. \end{array}$$

Now we have

(20) δd(xmk,xnk)≤δd(xmk,xmk+1)+d(xmk+1,xnk+1)+d(xnk+1,xnk)=δd(xmk,xmk+1)+d(f(xmk),f(xnk))+d(xnk+1,xnk)=δd(xmk,xmk+1)+d(xnk+1,xnk)δd(f(xmk),f(xnk))≤δd(xmk,xmk+1)+d(xnk+1,xnk)δ∗d(f(xmk),f(xnk))≤δd(xmk,xmk+1)+d(xnk+1,xnk) ×[α(xmk,xnk)−1+δ∗]d(f(xmk),f(xnk))≤δd(xmk,xmk+1)+d(xnk+1,xnk)δβ(d(xmk,xnk))d(xmk,xnk)=δd(xmk,xmk+1)+d(xnk+1,xnk)+β(d(xmk,xnk))d(xmk,xnk).

This implies that

(21) d(xmk,xnk)≤d(xmk,xmk+1)+d(xnk+1,xnk) +β(d(xmk,xnk))d(xmk,xnk).

That is,

(22) d(xmk,xnk)−d(xmk,xmk+1)−d(xnk+1,xnk)d(xmk,xnk)≤β(d(xmk,xnk))<1.

On taking limit as *k* → *∞* and using (14) and (18), we get

(23) $$\begin{array}{c} \underset{k\to \infty }{\lim}\beta \left(d\left(x_{m_{k}},x_{n_{k}}\right)\right)=1. \end{array}$$

Since *β* ∈ *Υ*, we have lim⁡_*k*→*∞*_⁡*d*(*x*_*m*_*k*__, *x*_*n*_*k*__) = 0 which contradicts with (18). Therefore, {*x*_*n*_} is a Cauchy sequence. By the completeness of *X*, we get lim⁡_*n*→*∞*_ *x*_*n*_ = x* for some x* ∈ *X*. Since *f* is continuous,

(24) $$\begin{array}{c} x^{*}=\underset{n\to \infty }{\lim}x_{n}=\underset{n\to \infty }{\lim}f\left(x_{n+1}\right)=f\left(\underset{n\to \infty }{\lim}x_{n+1}\right)=f\left(x^{*}\right). \end{array}$$

That is, x* is a fixed point of *f* and thus the fixed point problem of *f* has a solution. This completes the proof.

In the next theorem, we omit the continuity hypothesis of *f* by adding some condition.

**Theorem 9**

Let (*X*, *d*) be a complete metric space and *f* : *X* → *X* an *α*-*β*-contraction mapping satisfying the following conditions:

- *f* is *α*-admissible;
- *α* is transitive;
- there exists *x*_0_ ∈ *X* such that *α*(*x*_0_, *f*(*x*_0_)) ≥ 1;
- if {*x*_*n*_} is a sequence in *X* such that *α*(*x*_*n*_, *x*_*n*+1_) ≥ 1 for all *n* ∈ *ℕ* and *x*_*n*_ → *x* ∈ *X* as *n* → *∞*, then *α*(*x*_*n*_, *x*) ≥ 1 for all *n* ∈ *ℕ*.

Then the fixed point problem of *f* has a solution; that is, there exists x* ∈ *X* such that x* = *f*(x*).

**Proof**

Following the proof of Theorem 8, we know that {*x*_*n*_} is a Cauchy sequence in the complete metric space *X*. Then, there exists x* ∈ *X* such that *x*_*n*_ → x* as *n* → *∞*.

On the other hand, from (10) and hypothesis (iv), we have

(25) $$\begin{array}{c} \alpha \left(x_{n},x^{*}\right)\geq 1,\;\forall n\in \mathbb{N}. \end{array}$$

Now, using the triangular inequality, (6), and (25), we get

(26) δd(x∗,f(x∗))≤δd(x∗,xn+1)+d(xn+1,f(x∗))=δd(x∗,xn+1)+d(f(xn),f(x∗))=δd(x∗,xn+1)δd(f(xn),f(x∗))≤δd(x∗,xn+1)δ∗d(f(xn),f(x∗))≤δd(x∗,xn+1) ×[α(xn,x∗)−1+δ∗]d(f(xn),f(x∗))≤δd(x∗,xn+1)δβ(d(xn,x∗))d(xn,x∗)≤δd(x∗,xn+1)+β(d(xn,x∗))d(xn,x∗)

for all *n* ∈ *ℕ*. It follows that

(27) d(x∗,f(x∗))≤d(x∗,xn+1)+β(d(xn,x∗))d(xn,x∗)<d(x∗,xn+1)+d(xn,x∗)

for all *n* ∈ *ℕ*. Letting *n* → *∞* in the above relation, we obtain that *d*(x*, *f*(x*)) = 0; that is, x* = *f*(x*). Therefore, the fixed point problem of *f* has a solution. This completes the proof.

We obtain that Theorems 8 and 9 do not claim the uniqueness of fixed point. To assure the uniqueness of the fixed point, we will add some properties.

**Theorem 10**

Adding condition

(H_0_) *α*(*a*, *b*) ≥ 1 for all *a*, *b* ∈ *X*, where *a*, *b* are fixed points of *f*

or

(H_1_) for all *x*, *y* ∈ *X*, there exists *z* ∈ *X* such that *α*(*x*, *z*) ≥ 1 and *α*(*y*, *z*) ≥ 1

to the hypotheses of Theorem 8 (resp., Theorem 9) one obtains uniqueness of the fixed point of *f*.

**Proof**

Suppose that x* and y* are two fixed points of *f*. If condition (H_0_) holds, then we get the uniqueness of the fixed point of *f* from (6). So we only show that the case of (H_1_) holds. From condition (H_1_), there exists *z* ∈ *X* such that

(28) $$\begin{array}{c} \alpha \left(x^{*},z\right)\geq 1,\;\;\alpha \left(y^{*},z\right)\geq 1. \end{array}$$

Since x*, y* are fixed points of *f* and *f* is *α*-admissible, from (28), we get

(29) $$\begin{array}{c} \alpha \left(x^{*},f^{n}\left(z\right)\right)\geq 1, \end{array}$$

(30) $$\begin{array}{c} \alpha \left(y^{*},f^{n}\left(z\right)\right)\geq 1 \end{array}$$

for all *n* ∈ *ℕ*. From (29) and (6), we have

(31) δd(x∗,fn+1(z))=δd(f(x∗),f(fn(z)))≤δ∗d(f(x∗),f(fn(z)))≤[α(x∗,fn(z))−1+δ∗]d(f(x∗),f(fn−1(z)))≤δβ(d(x∗,fn(z)))d(x∗,fn(z))

for all *n* ∈ *ℕ*. Therefore,

(32) d(x∗,fn+1(z))≤β(d(x∗,fn(z)))d(x∗,fn(z))<d(x∗,fn(z))

for all *n* ∈ *ℕ*.

Next, we claim that lim⁡_*n*→*∞*_⁡*d*(x*, *f* ^*n*^(*z*)) = 0. Assume on the contrary that,

(33) $$\begin{array}{c} 0<\underset{n\to \infty }{\lim}d\left(x^{*},f^{n}\left(z\right)\right)<\infty . \end{array}$$

Letting *n* → *∞* in (32), we get

(34) $$\begin{array}{c} \underset{n\to \infty }{\lim}\beta \left(d\left(x^{*},f^{n}\left(z\right)\right)\right)=1. \end{array}$$

Using the fact that *β* ∈ *Υ*, we obtain that

(35) $$\begin{array}{c} \underset{n\to \infty }{\lim}d\left(x^{*},f^{n}\left(z\right)\right)=0 \end{array}$$

which is a contradiction. Therefore, we can conclude that

(36) $$\begin{array}{c} \underset{n\to \infty }{\lim}d\left(x^{*},f^{n}\left(z\right)\right)=0 \end{array}$$

and thus

(37) $$\begin{array}{c} \underset{n\to \infty }{\lim}f^{n}\left(z\right)=x^{*}. \end{array}$$

Similarly, using (30) and (6), we get

(38) $$\begin{array}{c} \underset{n\to \infty }{\lim}f^{n}\left(z\right)=y^{*}. \end{array}$$

By the uniqueness of limit of the sequence {*f* ^*n*^(*z*)}, we have x* = y*. This completes the proof.

**Remark 11**

Since Geraghty-type contraction mapping is an *α*-*β*-contraction mapping, Geraghty's fixed point results [23] can be considered as a corollary of our main results. Also, the Banach contraction principle [24] can be derived from our main results.

## 3. Generalized Ulam-Hyers Stability, Well-Posedness, and Limit Shadowing Results through the Fixed Point Problems

For the beginning of this section, we give the notion of generalized Ulam-Hyers stability in sense of a fixed point problem and also give the notion of well-posedness and limit shadowing property for fixed point problem.

**Definition 12**

Let (*X*, *d*) be a metric space and *f* : *X* → *X* a mapping. The fixed point problem

(39) $$\begin{array}{c} x=f\left(x\right) \end{array}$$

is called generalized Ulam-Hyers stability if and only if there exists the function *ψ* : [0, *∞*)→[0, *∞*) which is increasing, continuous at 0 and *ψ*(0) = 0 such that for each *ε* > 0 and for each w* ∈ *X* which is an *ε*-solution of the fixed point equation (39), that is, w* satisfies the inequality

(40) $$\begin{array}{c} d\left(w^{*},f\left(w^{*}\right)\right)\leq \varepsilon , \end{array}$$

there exists a solution x* ∈ *X* of (39) such that

(41) $$\begin{array}{c} d\left(x^{*},w^{*}\right)\leq \psi \left(\varepsilon \right). \end{array}$$

**Remark 13**

If the function *ψ* is defined by *ψ*(*t*) = *ct* for all *t* ≥ 0, where *c* > 0, then the fixed point equation (39) is said to be Ulam-Hyers stable.

**Definition 14 (see [12])**

Let (*X*, *d*) be a metric space and *f* : *X* → *X* a mapping. The fixed point problem of *f* is said to be well posed if it satisfies the following conditions:

- *f* has a unique fixed point x* in *X*;
- for any sequence {*x*_*n*_} in *X* such that lim⁡_*n*→*∞*_⁡*d*(*x*_*n*_, *f*(*x*_*n*_)) = 0, one has lim⁡_*n*→*∞*_⁡*d*(*x*_*n*_, x*) = 0.

**Definition 15**

Let (*X*, *d*) be a metric space and *f* : *X* → *X* a mapping. We say that the fixed point problem of *f* has the limit shadowing property in *X* if, for any sequence {*x*_*n*_} in *X* satisfying lim⁡_*n*→*∞*_⁡*d*(*x*_*n*_, *f*(*x*_*n*_)) = 0, it follows that there exists *z* ∈ *X* such that lim⁡_*n*→*∞*_⁡*d*(*f* ^*n*^(*z*), *x*_*n*_) = 0.

Concerning the generalized Ulam-Hyers stability, well-posedness, and limit shadowing property of the fixed point problem for a self-map of a complete metric space satisfying the conditions of Theorem 10, we have the following results.

**Theorem 16**

Let (*X*, *d*) be a complete metric space. Suppose that all the hypotheses of Theorem 10 hold and additionally that *β*(0) = 0 and the function *ξ* : [0, *∞*)→[0, *∞*) is defined by *ξ*(*t*): = *t* − *β*(*t*) which is strictly increasing and onto. Then

- if *α*(*a*, *b*) ≥ 1 for all *a*, *b* which are an *ε*-solution of the fixed point equation (39), then the fixed point problem of *f* is generalized Ulam-Hyers stability.
- if *α*(x*, *x*_*n*_) ≥ 1 for all *n* ∈ *ℕ* such that {*x*_*n*_} is sequence in *X* in which lim⁡_*n*→*∞*_⁡*d*(*x*_*n*_, *f*(*x*_*n*_)) = 0 and x* is a fixed point of *f*, then the fixed point problem of *f* is well posed;
- if *α*(x*, *x*_*n*_) ≥ 1 for all *n* ∈ *ℕ* such that {*x*_*n*_} is sequence in *X* in which lim⁡_*n*→*∞*_⁡*d*(*x*_*n*_, *f*(*x*_*n*_)) = 0 and x* is a fixed point of *f*, then the fixed point problem of *f* has the limit shadowing property in *X*.

**Proof**

From the proof of Theorem 10, we obtain that *f* has a unique fixed point and so let x* be a unique fixed point of *f*.

From the hypothesis in (a), we claim that the fixed point problem of *f* is generalized Ulam-Hyers stability. Let *ε* > 0 and w* ∈ *X* be a solution of (40); that is,

(42) $$\begin{array}{c} d\left(w^{*},f\left(w^{*}\right)\right)\leq \varepsilon . \end{array}$$

It is obvious that the fixed point x* of *f* satisfies inequality (40). From hypothesis in (a), we get *α*(x*, w*) ≥ 1. Now we have

(43) δd(x∗,w∗)=δd(f(x∗),w∗)≤δd(f(x∗),f(w∗))+d(f(w∗),w∗)=δd(f(x∗),f(w∗))δd(f(w∗),w∗)≤δ∗d(f(x∗),f(w∗))δd(f(w∗),w∗)≤[α(x∗,w∗)−1+δ∗]d(f(x∗),f(w∗)) ×δd(f(w∗),w∗)≤δβ(d(x∗,w∗))d(x∗,w∗)δd(f(w∗),w∗)=δβ(d(x∗,w∗))d(x∗,w∗)+d(f(w∗),w∗)≤δβ(d(x∗,w∗))d(x∗,w∗)+ε.

This implies that

(44) $$\begin{array}{c} d\left(x^{*},w^{*}\right)\leq \beta \left(d\left(x^{*},w^{*}\right)\right)d\left(x^{*},w^{*}\right)+\varepsilon \end{array}$$

and then

(45) $$\begin{array}{c} d\left(x^{*},w^{*}\right)-\beta \left(d\left(x^{*},w^{*}\right)\right)d\left(x^{*},w^{*}\right)\leq \varepsilon . \end{array}$$

That is,

(46) $$\begin{array}{c} \xi \left(d\left(x^{*},w^{*}\right)\right)\leq \varepsilon . \end{array}$$

Therefore,

(47) $$\begin{array}{c} d\left(x^{*},w^{*}\right)\leq \xi^{-1}\left(\varepsilon \right). \end{array}$$

It is easy to see that *ξ* ^−1^ is increasing, continuous at 0 and *ξ* ^−1^(0) = 0. Consequently, the fixed point problem of *f* is generalized Ulam-Hyers stability.

Next, we prove that the fixed point problem of *f* is well posed under the assumption in (b). Let {*x*_*n*_} be sequence in *X* such that lim⁡_*n*→*∞*_⁡*d*(*x*_*n*_, *f*(*x*_*n*_)) = 0. From assumption, we get *α*(x*, *x*_*n*_) ≥ 1 for all *n* ∈ *ℕ*. Now, we obtain that

(48) δd(x∗,xn)≤δd(x∗,f(xn))+d(f(xn),xn)=δd(f(x∗),f(xn))+d(f(xn),xn)=δd(f(x∗),f(xn))δd(f(xn),xn)≤δ∗d(f(x∗),f(xn))δd(f(xn),xn)≤[α(x∗,xn)−1+δ∗]d(f(x∗),f(xn)) ×δd(f(xn),xn)≤δβ(d(x∗,xn))d(x∗,xn)δd(f(xn),xn)=δβ(d(x∗,xn))d(x∗,xn)+d(f(xn),xn)

for all *n* ∈ *ℕ*. This implies that

(49) d(x∗,xn)≤β(d(x∗,xn))d(x∗,xn)+d(f(xn),xn)<d(x∗,xn)+d(f(xn),xn)

for all *n* ∈ *ℕ*. Now we claim that lim⁡_*n*→*∞*_⁡*d*(*x*_*n*_, x*) = 0. Assume on the contrary that

(50) $$\begin{array}{c} 0<\underset{n\to \infty }{\lim}d\left(x_{n},x^{*}\right)<\infty . \end{array}$$

From (49), we get lim⁡_*n*→*∞*_⁡*β*(*d*(*x*_*n*_, x*)) = 1. Since *β* ∈ *Υ*, we obtain that lim⁡_*n*→*∞*_⁡*d*(*x*_*n*_, x*) = 0 which contradicts with (50). Therefore, we conclude that lim⁡_*n*→*∞*_⁡*d*(*x*_*n*_, x*) = 0 and so the fixed point problem of *f* is well posed.

Finally, we prove that *f* has a limit shadowing under assumption (c). Let {*x*_*n*_} be sequence in *X* such that lim⁡_*n*→*∞*_⁡*d*(*x*_*n*_, *f*(*x*_*n*_)) = 0. Similar to case (b), we get lim⁡_*n*→*∞*_⁡*d*(*x*_*n*_, x*) = 0. Since x* is a fixed point of *f*, we have

(51) $$\begin{array}{c} \underset{n\to \infty }{\lim}d\left(x_{n},f^{n}\left(x^{*}\right)\right)=\underset{n\to \infty }{\lim}d\left(x_{n},x^{*}\right)=0. \end{array}$$

Therefore, *f* has the limit shadowing property.

*Some Open Problems*

- In Theorem 10, can we replace conditions (H_0_) and (H_1_) by other conditions or more general conditions?
- In Theorem 16, can we drop some conditions in (a), (b), and (c)?
- In Theorem 16, can we prove other types of stability of fixed point problem?
- Can we extend the result in this paper to other spaces as cone metric space, complex valued metric space, partial metric space, *b*-metric space, and circular metric space?
